# Nudge and bias in subjective ratings? The role of icon sets in determining ratings of icon characteristics

**DOI:** 10.3758/s13428-022-01973-7

**Published:** 2022-10-11

**Authors:** Siné McDougall, Irene Reppa, Jacqui Taylor

**Affiliations:** 1 Psychology Department, Bournemouth University, Poole, UK; 2 School of Psychology, Swansea University, Swansea, UK

**Keywords:** Ratings bias, Icon ratings, Icon design and usability, Concreteness, Complexity, Visual appeal, Affect

## Abstract

Subjective ratings have been central to the evaluation of icon characteristics. The current study examined biases in ratings in relation to the context in which icons are presented. Context was manipulated between participants, with some groups rating icon sets with limited variability, and others rating icon sets with wide variability. It was predicted that the context created by the icon set would influence participants’ ratings; when the range of icons was limited, this would create bias given participants’ expectation that a full range of icon values was being presented. Six key icon characteristics were rated, which were visual (visual complexity, appeal), affective (valence, feelings), and semantic (concreteness, semantic distance). Some icon characteristics were susceptible to rating bias while others were not. Where subjective judgements were being made of visual icon characteristics (appeal/complexity) and highly concrete icons which were very pictorial, there was clear evidence of substantial bias in ratings. The same susceptibility to bias was not evident when ratings relied solely on learned semantic associations or were associated with the emotional attributions made to icons. The dynamic nature of the ratings bias was demonstrated when the rating context was changed without participants’ knowledge. When participants rated further blocks of icons providing a different range of the to-be-rated characteristic, this resulted in rapid and dramatic changes in rating behaviour. These findings demonstrate the need for representative sampling of icon characteristics to avoid ratings bias. Practically, this is important when determining the usability of newly designed icon sets in order to avoid over-valuing or under-valuing of key characteristics.

A persistent issue for designers and human factors specialists is how the icons, symbols, and signs used on all kinds of equipment in the environment around us and in everyday personal communication can be made as effective—or usable—as possible. Icon usability is typically evaluated by asking groups of potential users to rate the properties of small sets of newly designed icons, and such methods are advocated by organisations promoting usability and design standards (e.g. British Standards Institution, 1989, 2012, 2015, 2019; International Organization for Standardization, 2019).

The methods employed to obtain icon ratings have their origins in subjective rating norms developed for words and pictures (e.g. Barry et al., 1997; Dan-Glauser & Scherer, 2011; Ito, Cacioppo, & Lang, 1998; Paivio et al., 1968; Spreen & Schulz, 1966; Gilhooly & Logie, 1980; Souza et al., 2021; Snodgrass & Vanderwart, 1980; Snodgrass & Yuditsky, 1996). Similar norms are available for icon corpora (Forsythe et al., 2017; McDougall, & Curry, & de Bruijn, O., 1999; Prada et al., 2015; Rodrigues et al., 2018; Souza et al., 2020). They include measures associated with ease of access to meaning such as meaningfulness, concreteness, name agreement, and ambiguity, as well as measures of the goodness of fit between the icon and its intended meaning such as semantic distance, name agreement, and clarity/ambiguity. Other measures are more perceptually based, such as visual complexity and aesthetic appeal. The affective response which icons elicit, such as valence (feelings of positivity vs negativity towards the icon) and arousal (whether or not an icon is calming or exciting) are increasingly considered to be important, particularly where icons will appear on consumer or social media interfaces (see Souza et al., 2020, for a review).

In addition to assessing potential usability, icon ratings have been used for experimental control in research. In early studies examining how icon characteristics affected user performance, experimenters used their own judgements to categorise icon characteristics, rather than employing participants’ subjective ratings. This sometimes resulted in unexpected confounds. For example, comparisons between the effects of concrete versus abstract icons on performance were often confounded by the fact that the concrete icons were more pictorial and so tended to contain more visual detail, thus confounding visual complexity and concreteness (Arend et al., 1987; Green & Barnard, 1990; Rogers, 1986; Stammers et al., 1989). Subsequent research, using subjective ratings to control concreteness and complexity, was able to show that visual complexity and concreteness have quite different effects on user performance and thus need to be considered separately (Isherwood et al., 2007; McDougall et al., 2000, 2001; Reppa et al., 2008; Reppa & McDougall, 2015). Subjective ratings have therefore become an important tool in measurement and experimental control of icon properties.

## Nudge and bias in rating icon sets

Despite the potential advantages of using subjective ratings, there has always been concern about the reliability of subjective reports and rating judgements. In one of the earliest studies of subjective reports, Galton (1883) noted that he had ‘little doubt that there is an unconscious exaggeration in these returns [subjective reports]’ (p. 66; see also Burbridge, 1994). Some researchers were wary of assuming that subjective ratings provided a free pass to good experimental control, given the biases they observed in subjective judgements, even going as far as suggesting that obtaining ‘measures from human judgements is a pointless exercise’ (Forsythe et al., 2017, p. 1484; Forsythe et al., 2008). For example, Forsythe et al. (2008) found that subjective ratings of visual complexity changed as users became more familiar with the icons in a set: complex icons were gradually perceived as simpler as the icons were learned and became more familiar. To resolve this issue, they proposed the use of automated measures of complexity such as using the file sizes of the GIF and JPEGs of the icons as an alternative to ratings. Metrics are unlikely to be appropriate for other icon characteristics such as visual appeal, valence, arousal, or semantic distance. As a result, researchers and icon designers alike need to have a good understanding of potential sources of bias that may result from having the human rater in the loop when subjective ratings are employed as a measure of icon characteristics.

While researchers, particularly of psycholinguistic stimuli, are aware of the need to provide large representative sets for evaluation (see Brysbaert, 2007; Clark, 1973; Raaijmakers, 2003; Raaijmakers et al., 1999), awareness of this requirement is sometimes lost, particularly when new icon sets are being evaluated for practical application where resources and time for measurement of key icon characteristics may be limited. The danger when carrying out design and usability evaluations of new icon sets for practical application is that the sets chosen for evaluation are those regarded as the best fit for subsequent use, with icons outside the newly created set rarely used as distractors. This means that newly created icon sets are often likely to have limited variability and this may systematically bias the subjective ratings obtained when evaluating their usefulness. Such a possibility is given credence not only because of Forsythe et al.’s research, but also because of the wealth of recent evidence which shows that individual judgements and decisions can often be unconsciously changed, or biased, by the way in which choices are presented.

The way that choices are presented—known as choice architecture—can create choice environments which exert an unconscious influence on individuals, nudging them towards certain decisions about purchasing, health and well-being, and finance by creating choice environments which exert an unconscious influence on the judgements individuals make (Benaratzi et al., 2017; Johnson et al., 2012; Thaler & Sunstein, 2008; Weinmann et al., 2016). We propose that presenting participants with a limited range of values for a to-be-rated icon characteristic, as is likely to be the case when small sets of novel icon sets are evaluated, unintentionally creates a choice architecture which nudges the subjective judgements participants make when rating the icons in the sets, creating bias. If participants assume that they are seeing the full range of variability for a given icon characteristic, ratings are likely to change accordingly.

### Experiments and hypotheses

The current series of experiments were conducted to examine the extent to which the range of values in the icon set to be rated creates a context which acts as an unconscious nudge for participants creating bias in icon ratings. To our knowledge, this is the first study examining the possibility that participants’ ratings may change relative to the range of icon values presented for the to-be-rated characteristic. Six experiments examined participants’ subjective ratings of visual, affective, and semantic icon dimensions. Two characteristics were selected for the perceptual (visual complexity and appeal), affective (valence and feelings), and semantic characteristics (concreteness and semantic distance) of the icons to be rated. Further details about each icon characteristic and why they were selected is included in the Materials section. Conditions were identical across all six experiments.

**Fig. 1:**
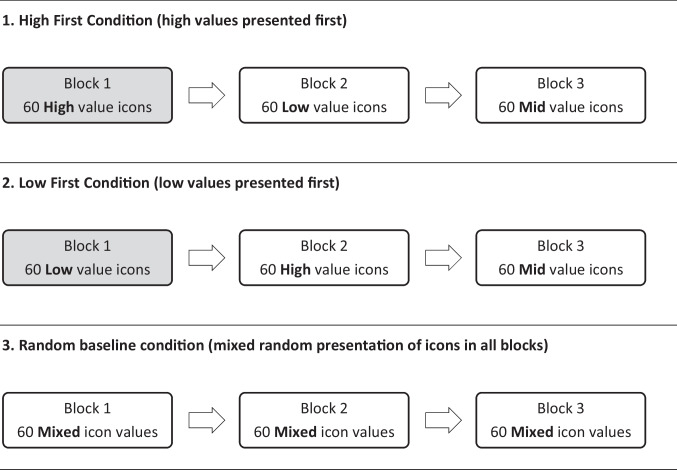
Order of presentation of icon type in each block of icons for the three experimental conditions

Figure 1 shows the order of icon presentation in each condition. In each experiment, participants were presented with 180 icons in blocks of 60 icons each in one of three conditions:

(i) The High First condition
  In the first block of 60, icons had *high* values on the icon characteristic of interest (for example, a set of highly visually appealing icons). In the second block icons had *low* values (e.g. unappealing icons), and in the third block icons with *mid-level* values were presented (moderately appealing icons; B1 high –> B2 low –> B3 mid-value blocks).
(ii) The Low First condition
  In this condition, the same icons were presented but the order of presentation was changed. The *low*-value icon block was presented first, followed by the *high*-value block, and then the *mid*-level block (B1 low –> B2 high –> B3 mid-value).
(iii) The Random Baseline condition
  Exactly the same icons were employed as in the experimental conditions, but presentation was randomised across all 180 icons, and participants experienced the full range of values of the icon characteristic being rated in all three blocks of 60 icons.

Participants were never informed about blocked presentation, and there was no demarcation to indicate that different blocks of icons were being presented.

It is important to note that the icons in the low-, mid-, or high-value categories were determined by a series of pilot studies for each individual characteristic. As a result, the icons in each of the three categories differed depending on the icon characteristic being examined in that experiment (see Table 7 for examples of each type of icon).

#### Hypothesis 1: In Block 1, a limited range of icon values creates ratings bias given participants’ expectation that a full range of icon values is presented

**Fig. 2:**
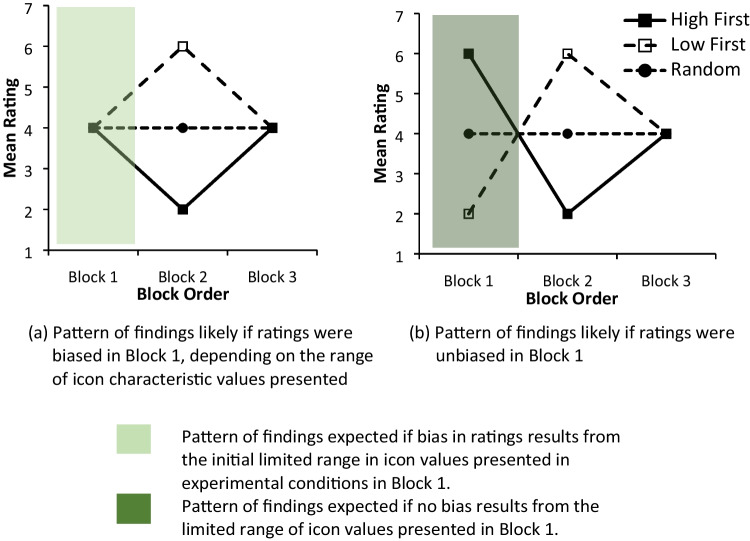
Possible patterns of subjective ratings depending on whether the range of icons presented in the rating set created bias in the ratings obtained in Block 1 and recalibration of ratings in Block 2

In Block 1, the High First and Low First experimental conditions mimicked the scenario where newly designed icons which are limited in their range of values on a given icon characteristic are presented for rating evaluation. The possible patterns of ratings are shown in the light and dark green highlighted areas in Fig. 2a and b, respectively. In the High First condition, participants might be expected to employ ratings of mainly 6 or 7 given that only high values of an icon characteristic are presented. Hypothesis 1 proposes that where an icon set is limited in range, participants will, nevertheless, provide ratings across the *full range* of the rating scale they are given and so use *all ratings* on the scale, from 1 to 7. The same principle applies in the Low First condition. Rather than ratings of primarily 1 or 2 to indicate low values on a given characteristic, the full range of ratings, i.e. 1–7, would be used. In Block 1 this would produce similar mean ratings of approximately 4 in both the High First and Low First conditions, despite the very different nature of the high- and low-value icons presented (see Fig. 2a). Participants in the Random Baseline condition, who are shown the full range of icon values, would similarly produce a mean rating of approximately 4. Thus, the expectation to employ the full range of the rating scale, even when only high-value or only low-value icons are presented, creates a ratings bias which leads to a similar range of ratings being obtained across *all* three *conditions* despite the range of to-be-rated icons differing considerably. The alternative to this predicted pattern of ratings, shown in Block 1, Fig. 2b, is that participants produce high ratings in the High First condition, low ratings in the Low First condition, and mid-range values in the Random Baseline condition.

#### Hypothesis 2: In Block 2, changing the icon values presented in the experimental conditions provides a counter-nudge to participants, producing a recalibration in participant ratings

In Block 2 there is a step change in the nature of the icons presented for rating in the experimental conditions (from high –> low in the High First condition and low –> high in the Low First condition; see Fig. 1). If ratings bias has occurred in Block 1, it was expected that the change in icon values would provide a crucial comparator, or *counter-*nudge, cuing participants to recalibrate their ratings to accommodate the wider range of icon values presented. If effective, this counter-nudge would produce much *lower* ratings at Block 2 for participants in the High First condition (as participants moved from high –> low icon values) and much *higher* ratings in the Low First condition (as participants moved from low –> high icon values). This would occur even though participants would not be told that they were being presented with blocks of icons or that those icons changed in their nature from one block to the next. Ratings in the Random Baseline condition were, of course, expected to remain the same throughout all blocks. If recalibration of ratings *does not occur* in Block 2, then the pattern of ratings would remain similar to that obtained in Block 1.

In Block 3, the remaining 60 icons with mid-level values are presented in High First and Low First conditions. In this way participants, irrespective of whether they were in the experimental or Random Baseline conditions, would have experience of the full range of icon values and so mean ratings were expected to return nearer to the mid-range in the final block of icons.

To summarise, participants’ ratings of icon characteristics are often used in both applied and research settings to evaluate comprehensibility and aesthetic and affective responses to novel icon sets. This study sought to examine potential bias in subjective ratings which may arise when the ratings context was changed and participants were asked to evaluate sets of stimuli with a limited range of values. Bias was expected to result from participants’ expectation that they would use the full range of ratings across a scale producing markedly different patterns of rating as a result.

## Method

### Participants

Prior norming studies of icon corpora have employed participant samples of between 30 and 49 for each icon characteristic of interest (McDougall, & Curry, & de Bruijn, O., 1999; Prada et al., 2015; Rodrigues et al., 2018). These studies do not include between-group comparisons at different time points, and to date there are no comparable studies on which sample sizes might be based. A priori power analyses were therefore employed using G*Power to calculate appropriate sample sizes (G*Power 3.1.9.2, Faul et al., 2007). When a medium effect size was assumed with α = 0.05 and 1 − β = 0.80, a total sample size of *n* = 42 participants was estimated; the sample size estimate increased to *n* = 60 when 1 − β = 0.95. A total of 473 participants took part in the six experiments reported. Twenty-nine participants were excluded from the study when they failed to complete 90% of the ratings survey, leaving a total of 444 participants. The total number of participants in each experiment ranged from 67 to 82. Table 1 shows the number, age, and gender of participants in each condition. In addition, 6–14 participants were recruited for each pilot study to provide norms determining high-, low-, and mid-value icons for each characteristic (see details in Materials section).

**Table 1:** Biographical information obtained from participants in Experiments 1–6: age, gender, frequency of use of IT, and overall rating of expertise

Nature of information obtained	Group	Experiment 1 Appeal ratings	Experiment 2 Complexity ratings	Experiment 3 Valence ratings	Experiment 4 Feelings ratings	Experiment 5 Concreteness ratings	Experiment 6 Semantic distance ratings
Age (mean, SD)	High First	19.62 (1.42)	21.04 (4.96)	23.50 (6.83)	20.83 (2.76)	22.77 (6.68)	20.27 (2.57)
	Low First	20.11 (3.56)	20.85 (3.76)	22.15 (6.93)	20.71 (2.85)	19.13 (1.96)	20.05 (2.48)
	Mid-Level	21.19 (3.83)	19.79 (3.67)	22.43 (6.36)	20.14 (1.75)	20.86 (0.90)	20.10 (2.38)
	Total	20.04 (2.66)	20.54 (4.17)	22.73 (6.67)	20.58 (2.54)	20.89 (5.00)	20.15 (2.45)
No. in each group (number female^+^)	High First	47 (41 F)	24 (22 F)	28 (24 F)	28 (15 F)	22 (16 F)	26 (24 F
	Low First	19 (15 F)	20 (18 F)	26 (23 F)	31 (30 F)	23 (15 F)	21 (17 F)
	Mid-Level	16 (14 F)	24 (21 F)	23 (19 F)	22 (18 F)	29 (23 F)	20 (19 F)
	Total	82 (70 F)	68 (61 F)	77 (66 F)	76 (63 F)	74 (54 F)	67 (60 F)
% Participants who were frequent users of mobiles and tablets in each group*	High First	95.74	100.00	100.00	100.00	100.00	100.00
	Low First	100.00	100.00	100.00	93.55	100.00	100.00
	Mid-Level	100.00	100.00	95.65	100.00	100.00	100.00
	Total	98.50	100.00	98.55	97.85	100.00	100.00
% Participants who were frequent users of laptops and PCs in each group*	High First	82.97	79.16	92.86	95.65	86.36	96.15
	Low First	89.47	85.00	96.15	93.55	91.30	100.00
	Mid-Level	87.50	100.00	82.16	95.45	86.21	85.00
	Total	86.60	88.05	90.39	94.88	87.84	94.03
Overall rating of expertise^**^ (mean, SD)	High First	5.14 (0.84)	5.58 (1.06)	5.71 (1.05)	5.09 (1.04)	5.14 (1.21)	5.54 (0.95)
	Low First	5.16 (0.37)	5.15 (1.63)	5.62 (0.85)	5.32 (1.04)	5.09 (1.16)	4.86 (0.96)
	Mid-Level	4.79 (1.19)	5.29 (1.23)	5.87 (0.87)	5.50 (0.91)	5.24 (1.06)	5.85 (0.99)
	Total	5.08 (0.84)	5.35 (1.30)	5.73 (0.93)	5.30 (1.01)	5.16 (1.12)	5.42 (1.03)

**+**Participants were offered the opportunity to define gender via free text, but none did so. *Frequent use was defined as between 4 and 7 times a week **Participants used a 1–7 rating scale with high ratings indicating higher perceived level of expertise

This research was carried out in accordance with the Declaration of Helsinki, and ethics approval was obtained at the universities of each of the authors (reference nos. 34784, 52584374 and H13946; reference numbers are in the same order as authors). Before taking part, all participants were informed about the nature of the study and gave their informed consent. Participants were recruited from all three universities and were mainly undergraduate psychology students. Some participants in pilot studies were also recruited via personal connections with the researchers. While the predominance of females in the sample was not ideal, previous research which examined gender differences in rating icons have consistently found null results (McDougall, & Curry, & de Bruijn, O., 1999; Prada et al., 2015; Rodrigues et al., 2018). Participants were additionally asked about their frequency of use of mobile phones, tablets, laptops, or computers and their perceived overall expertise in using information technology. Almost all participants were frequent users of information technology and considered themselves to be relatively expert users.

### Selection of icon characteristics

In a recent review, Souza et al. (2020) argued that a combination of perceptual, affective, and semantic dimensions should be considered, highlighting the increasing weight of evidence arguing that processing of pictorial stimuli involved all three factors (see also Kensinger, 2007; Konkle et al., 2010; Li et al., 2020). Icon-specific research has also demonstrated the importance of these factors in determining user performance (Forsythe et al., 2017, Forsythe et al., 2008; Isherwood et al., 2007; McDougall et al., 2000; Reppa, McDougall, Sonderegger, & Schmidt, 2021; Reppa & McDougall, 2015). This is also reflected in practice. Initially, evaluation of novel icon sets focused on semantics by ensuring the comprehensibility of icons, but as consumer websites and social media have assumed greater importance, the visual appeal and affective responses to icons have also become a focus in selecting icon sets. Six icon characteristics were selected for examination, with two characteristics chosen to represent each of the three key dimensions:

(i) Perceptual: Visual appeal and visual complexity
(ii) Affective: Valence and feelings
(iii) Semantic: Concreteness and semantic distance.

This allowed us to examine the possibility that contextual bias in rating, if it occurred, might either depend on the rating *dimension* or be specific to individual *icon characteristics*. Familiarity was not selected for consideration, not least because there are several potential sources of familiarity for each icon: familiarity with what is depicted in the icon, familiarity with the intended meaning, and familiarity with the association between depiction and meaning (see McDougall & Isherwood, 2009, for further discussion of this issue). The role each icon characteristic plays in cognitive processing and its import for usability is now considered.

#### Visual appeal (Experiment 1)

Visual appeal is an integral part of our initial apprehension of objects and scenes and is discerned extremely quickly (Lindgaard et al., 2006; Lindgaard et al., 2011; Thielsch et al., 2014; Thielsch & Hirschfeld, 2019). Visual search is faster when icons and interfaces are appealing (Moshagen et al., 2009; Reppa & McDougall, 2015; Sonderegger & Sauer, 2010). Recent research suggests that appealing stimuli are inherently rewarding and so produce better performance in what might otherwise be difficult conditions (Reppa et al., 2021).

#### Visual complexity (Experiment 2)

Visual complexity is one of the most frequently examined characteristics of icons and pictures (Souza et al., 2020) and is typically implicated in visual search. Icon complexity has a negative influence on icon search performance (Byrne, 1993; Gerlach & Marques, 2014; Isherwood et al., 2007; McDougall et al., 2000; McDougall et al., 2006; Reppa et al., 2008; Reppa & McDougall, 2015; Scott, 1993). Icon complexity may have particularly detrimental effects on search when task demands are high (Gerlach & Marques, 2014) or when carrying out visual search at times of day when performance is often poorer (e.g. during the ‘post-lunch dip’; McDougall et al., 2006).

#### Valence (Experiment 3)

Positive regard for icons has been evaluated by obtaining subjective ratings of users’ affective responses. Valence (positive/pleasant vs negative/unpleasant) has been regularly included as a rating characteristic for words, pictures, symbols, and icons (Ito, Cacioppo, & Lang, 1998; Libkuman et al., 2007; Moors et al., 2013; Prada et al., 2015; Rodrigues et al., 2018; Söderholm et al., 2013; Warriner et al., 2013). The precise role of valence in determining user performance is unclear, but it is widely implicated in cognition including perception, working memory, and semantic activation (Angrilli et al., 1997; Galindo et al., 2015; Montoya et al., 2017; Pauligk et al., 2019; Storbeck & Clore, 2007; Warrington & Shallice, 1984; Yamaguchi & Chen, 2018; Zdrazilova & Pexman, 2013).

#### Feelings (Experiment 4)

Arousal ratings (calm/passive vs arousing/exciting) have often been used as another index of emotional responsivity to stimuli (Dan-Glauser & Scherer, 2011; Moors et al., 2013; Söderholm et al., 2013; Stadthagen-Gonzalez, Imbault, Pérez Sanchez, & Brysbaert, 2015; Warriner et al., 2013). In the present study, however, participants were asked about their feelings in a way that might make contact more directly with the ‘emotions’ likely to be elicited by icons where emotional responses are likely to be less intense. Participants were asked to consider the extent to which an icon made them feel cheery or depressed, with 1 = very depressing and 7 = very cheery as scale anchors. This scale was an amended form of a cheerfulness scale employed by Raymond et al. when examining affective responses to complex colourful images in a visual search task (see Fenske et al., 2004; Raymond et al., 2003).

#### Concreteness (Experiment 5)

Concreteness has been at the heart of the semantic evaluation of icons. Concrete icons are thought to be easier to use and learn because they depict objects and people we are familiar with on the basis of our real-world knowledge. Simply naming what is depicted in a concrete icon can often allow us to infer its meaning even if we have not encountered it before. In contrast, for abstract icons, where meaning is represented using shapes, arrows, and so on, meaning must be inferred if possible (see Table 2, icons a vs d) or may be known as a result of semantic associations learned over time (Table 2, icon c). Accuracy and speed of icon identification is better when icons are concrete (e.g. Chan & Ng, 2010; Green & Barnard, 1990; Leung et al., 2011; McDougall et al., 2000; Rogers & Oborne, 1987; Schroder & Ziefle, 2008; Stotts, 1998), and concreteness is a particularly strong determinant of performance when icon sets are new to users, or are used sporadically, but becomes less important once icons are familiar (Isherwood et al., 2007; McDougall et al., 2000, 2001).

**Table 2:**
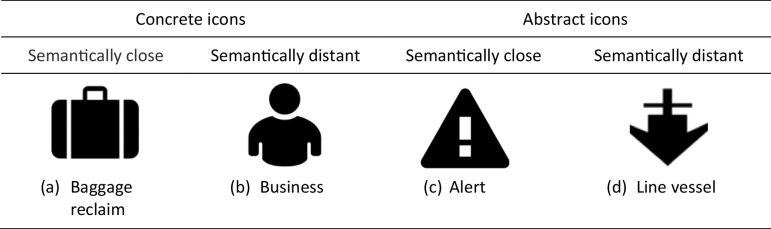
Examples of icons differing in concreteness and semantic distance

#### Semantic distance (Experiment 6)

Semantic distance measures the strength of the association between what is depicted in an icon and the meaning it is intended to convey. It is arguably a better index of icon meaningfulness. This is because, as the number of icon functions we wish to represent grows, it becomes harder to find concrete depictions which have a simple fit to the intended meaning. This is illustrated in Table 2, which shows that both concrete and abstract icons can have a good or poor fit between what is depicted and the intended meaning (Table 2a vs b and c vs d). Semantic distance is similar to the measure of picture–name agreement employed by Souza et al. (2020) for real-world pictures, where participants were asked to evaluate the extent to which the image corresponded to the name presented, and to the measure of ‘goodness of depiction’ employed by Bates et al. (2003) where participants were asked to rate how well the picture fitted its dominant name. In Bates et al.’s (2003) study of predictors of picture naming latency across seven languages, goodness of depiction was found to be the most important predictor of picture naming latency. Similarly, research to date using icons suggests that semantic distance is more important than concreteness in determining participants’ icon identification performance (Isherwood et al., 2007; McDougall et al., 2001; McDougall & Isherwood, 2009).

### Materials

A set of 300 icons were selected from a wide variety of sources in order to ensure that they were representative of the broad spectrum of applications in which icons, symbols, and signs are currently used and which provided a full range of variation in each of the icon characteristics to be tested. Icons included public information signs and symbols used on electrical and medical equipment, on vehicle controls, and on computers, tablets, and mobile phones. These included symbols from the International Organization for Standardization and International Electrotechnical Commission, as well as UK government information signs and a variety of icons from free-to-use sites. The full set of icons and their sources is available via the following link: 10.6084/m9.figshare.19550833.

#### Pilot studies

High, low, and mid-level values for each icon characteristic were determined by a series of pilot studies carried out prior to each experiment, in which participants rated all 300 icons, which were presented in random order. Separate participant groups were used for each icon characteristic so that each participant rated only one icon characteristic in order to avoid participant fatigue and ratings being affected by growing familiarity with items if rated repeatedly on different dimensions (see Forsythe et al., 2017). Table 3 shows the number, age, and gender of participants in each condition. Table 4 provides the summary statistics for the icon, symbol, and sign characteristics of the icon corpus. Although mean and median values differed between stimulus characteristics, ratings were distributed relatively normally, with low levels of skew.

**Table 3:** Biographical information obtained from pilot study participants: age, gender, frequency of use of IT, and overall rating of expertise

Nature of information obtained	Experiment 1 Appeal ratings	Experiment 2 Complexity ratings	Experiment 3 Valence ratings	Experiment 4 Feelings ratings	Experiment 5 Concreteness ratings	Experiment 6 Semantic distance ratings
Age (mean, SD)	49.83 (22.82)	22.87 (1.64)	19.75 (0.88)	26.92 (12.01)	54.00 (15.07)	19.28 (1.59)
No. in each group (number of females^+^)	6 (4F)	11 (9F)	8 (7F)	13 (11F)	7 (5F)	14 (13F)
% Participants who were frequent users of mobiles and tablets in each group*	66.67	100.00	100.00	100.00	100.00	100.00
% Participants who were frequent users of laptops and PCs in each group*	83.33	63.63	51.14	76.92	71.42	100/00
Overall rating of expertise^**^ (mean, SD)	5.50 (1.64)	5.72 (1.64)	5.00 (0.93)	5.69 (0.75)	4.43 (1.29)	5.00 (0.88)

^**+**^Participants were offered the opportunity to define gender via free text, but none did so. *Frequent use was defined as between 4 and 7 times a week **Participants used a 1–7 rating scale, with high ratings indicating higher perceived level of expertise

**Table 4:** Summary statistics of icon, symbol, and sign characteristics from pilot study (N = 300)

Characteristics	Mean	Median	SD	Min	Max	Skew	Kurtosis
1. Appeal	3.04	2.83	1.09	1.17	6.00	0.441	−.522
2. Complexity	3.33	3.25	1.04	1.13	6.13	0.473	−.176
3. Concreteness	4.23	4.24	1.60	1.20	7.00	0.071	−1.149
4. Semantic distance	4.60	4.78	1.68	1.64	6.93	0.187	−1.435
5. Valence	4.12	4.00	0.92	1.63	6.75	0.269	−.020
6. Feelings	3.98	3.86	0.76	1.93	6.43	0.521	−.159

Table 5 shows the correlations between icon characteristics. The pattern of correlations is similar to those obtained in recently reported corpora which employed repeated-measures designs (i.e. participants rated all icon characteristics; Prada et al., 2015; Rodrigues et al., 2018). Correlations were lowest between icon complexity and other icon characteristics, particularly appeal (*r* = −.111), and highest between indices of affect (*r* = .777 between valence and feelings) and meaning (*r* = .710 between concreteness and semantic distance; see Prada et al., 2015; Rodrigues et al., 2018, for similar patterns of correlations).

**Table 5:** Correlations between icon, symbol, and sign characteristics from pilot study (N = 300)

Characteristics	1	2	3	4	5
1. Appeal	-				
2. Complexity	−.111	-			
3. Concreteness	.676**	−.098	-		
4. Semantic distance	.555**	−.126*	.710**	-	
5. Valence	.441**	−.142*	.388**	.326**	-
6. Feelings	.617**	−.057	.536**	.452**	.777**

**Correlation is significant at the .01 level (two-tailed)

*Correlation is significant at the .05 level (two-tailed)

Three sets of 60 icons were selected for each of the six experiments: the 60 icons with the highest ratings, the 60 with the mid-level ratings, and the 60 with the lowest ratings. Summary statistics for low-, mid-, and high-value icons in each condition are shown in Table 6. A by-item one-way analysis of variance followed by Newman–Keuls comparisons revealed reliable differences between each set of 60 icons. Table 7 shows examples of the low-, mid-, and high-value icons used in each experiment. Because low, mid, and high values were determined by individual pilot studies for each icon characteristic, it was possible for some icons to be presented in different value categories in different experiments depending on the icon characteristic; for example, ‘pixel averaging’ appears in the low-value condition for semantic distance but in the mid-level condition for valence.

**Table 6:** Summary statistics of icons, symbols, and signs for low, mid, and high values (N = 60 in each condition) along with the results of the one-way analysis of variance and Newman–Keuls comparisons carried out to examine differences between icon ratings in the low-, mid-, and high-value icons

Stimulus characteristic	Icon type	Mean	SD	Min	Max	*F* value	Newman–Keuls comparisons
E1: Appeal	Low	1.66	0.28	1.17	2.00	*F*(2,177) = 1372.61, *p* < .001	High>Middle>Low
	Mid	2.91	0.16	2.67	3.17		
	High	4.73	0.46	4.00	6.00		
E2: Complexity	Low	1.98	0.34	1.13	2.38	*F*(2,177) = 864.97, *p* < .001	High>Middle>Low
	Mid	3.26	0.15	3.21	3.29		
	High	4.89	0.55	4.26	6.13		
E3: Valence	Low	2.94	0.44	1.63	3.38	*F*(2,177) = 806.87, *p* < .001	High>Middle>Low
	Mid	4.01	0.11	3.75	4.25		
	High	5.53	0.41	4.88	6.75		
E4: Feelings	Low	3.01	0.24	1.93	3.29	*F*(2,177) = 890.53, *p* < .001	High>Middle>Low
	Mid	3.82	0.11	3.64	4.00		
	High	5.11	0.39	4.64	6.43		
E5: Concreteness	Low	1.99	0.40	1.20	2.71	*F*(2,177) = 2413.87, *p* < .001	High>Middle>Low
	Mid	4.28	0.33	3.80	4.80		
	High	6.39	0.31	5.86	7.00		
E6: Semantic distance	Low	2.21	0.28	1.64	2.86	*F*(2,177) = 2455.83, *p* < .001	High>Middle>Low
	Mid	4.77	0.51	3.86	5.50		
	High	6.66	0.18	6.36	6.93

**Table 7:**
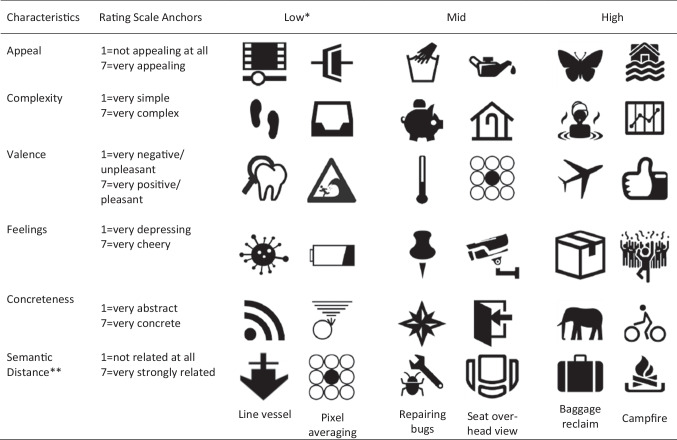
Examples of icons with low, mid, and high rating values for each icon characteristic

* Icons with the 60 lowest, 60 highest, and 60 mid-rating values in the pilot study for each icon characteristic

** Icons were shown without the meaning they were intended to convey, with the exception of meaning/semantic distance ratings, where participants were asked to indicate the goodness of fit between the icon and its given meaning

### Procedure

Data were gathered online using Qualtrics software (Qualtrics, Provo, UT, USA). Participants were invited to take part either via the universities’ participant recruitment systems or via an email containing the hyperlink for the ratings questionnaire. The rating task began immediately following participants’ indication that they had read the experimental information and consented to take part. The procedure followed by participants in Experiments 1–6 was identical, with the exception of the rating instructions given. Table 8 shows the rating instructions given to participants prior to starting the rating and for each icon characteristic along with the Likert scale anchor points.

**Table 8:** Instructions and scale anchors for each icon characteristic

Characteristics	Instructions	Scale
Appeal	Please indicate how visually appealing or unappealing each icon is.	1 = not appealing at all 7 = very appealing
Complexity	Please consider the amount of visual detail or intricacy in each of the icons you are shown, then indicate how simple or complex each one is.	1 = very simple 7 = very complex
Concreteness	Please indicate how concrete or abstract each icon is: ▪ Concrete icons contain or show real things*, people, or materials. ▪ Abstract icons *do not contain or show real things*, people, or materials.	1 = very abstract 7 = very concrete
Semantic distance**	Please indicate how well you think each icon fits with the meaning it is intended to convey.	1 = not related at all 7 = very strongly related
Valence	Please indicate the extent to which you consider the icons to refer to something positive/pleasant or negative/unpleasant.	1 = very negative/ unpleasant 7 = very positive/ pleasant
Feelings	Please indicate whether an icon cheers you up or makes you feel a bit depressed.	1 = very depressing 7 = very cheery

*Italics used in this table were not used in instructions given to participants

**Icons were shown with the intended meaning only for ratings of semantic distance, where participants were asked to indicate the goodness of fit between the icon and its given meaning

Instructions were similar to those employed in previous research for appeal (McDougall & Reppa, 2008; Prada et al., 2015), visual complexity (McDougall, & Curry, & de Bruijn, O., 1999; Snodgrass & Vanderwart, 1980), concreteness (for icons: McDougall, & Curry, & de Bruijn, O., 1999; Prada et al., 2015; forwards: Gilhooly & Logie, 1980; Paivio et al., 1968; Spreen & Schulz, 1966), semantic distance (Isherwood et al., 2007; McDougall, & Curry, & de Bruijn, O., 1999), valence (Moors et al., 2013; Prada et al., 2015), and feelings (an amended scale with depressing/cheery as anchors, see Raymond et al., 2003; Fenske et al., 2004).

All participants were asked to consider each icon before rating as follows:

‘Please consider each icon before rating it but do not spend too long on each one—it is your first impression that is important. There is no wrong or right answer—it is your unique view that counts, so please do not ask others what their opinion is.’

Participants were shown icons in three blocks with 60 icons in each block; 12 icons were presented on each survey page, with five pages in each block. Participants only rated one icon characteristic in one experimental condition, because (a) naïveté about block order was of critical importance to the experimental hypotheses, and (b) it was assumed that rating on one characteristic might affect subsequent ratings of other icon characteristics.

## Results

### Statistical analysis

**Fig. 3:**
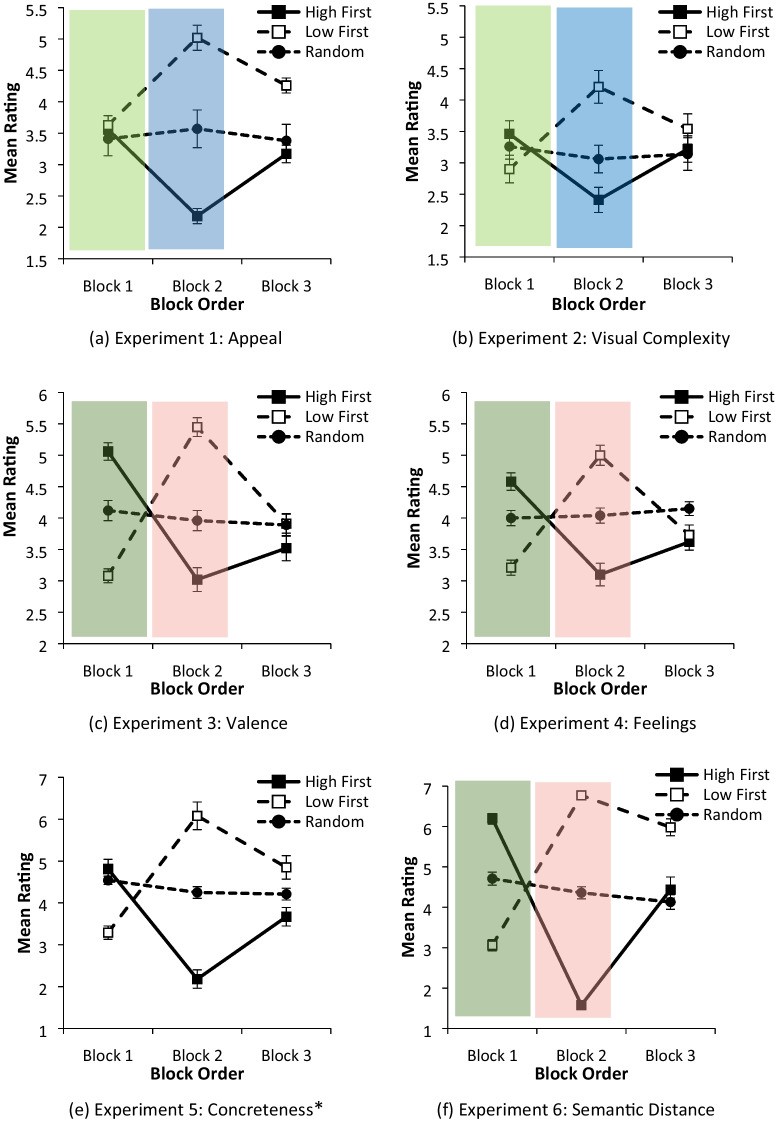

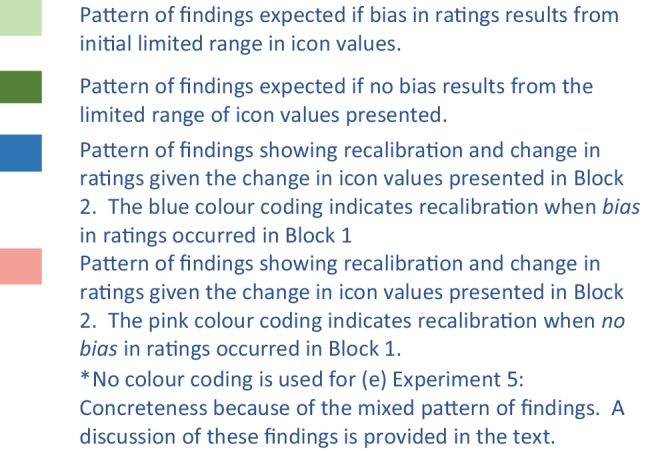
Mean and standard errors of subjective ratings obtained for visual (appeal, visual complexity), affective (valence, feelings), and semantic (concreteness, semantic distance) characteristics of icons

Data from the pilot and experimental studies are available at the following link 10.6084/m9.figshare.19550833. Each experiment was analysed using a 3 Condition (High/Low/Random baseline) × 3 Block (Block 1/2/3) analysis of variance in which Condition was between subjects and Block was within subjects. Where Mauchly’s test of sphericity was significant, the Greenhouse–Geisser correction was reported for within-subject effects. Bonferroni pairwise comparisons were used to examine differences between conditions for each icon block. Data from each experiment were analysed separately, rather than in an omnibus analysis for all six experiments, because the icons appearing as high, low, and mid-level values of each icon characteristic depended on the ratings obtained in six individual pilot studies and therefore differed between experiments. The results of these analyses are summarised in Table 9. The pattern of findings for each experiment is illustrated in Fig. 3 and can be compared with the predicted pattern of findings in Fig. 2.

**Table 9:** Experiments 1–6: Outcome of 3 Condition (High/Low/Random) × 3 Block Order (1/2/3) mixed analyses of variance with repeated measures on Block Order carried out on the subjective rating data. F-values, p-values, and ηp^2^ are shown for the main effects and interaction term along with the p-values for Bonferroni pairwise comparisons between conditions in each block

Stimulus characteristic	Condition	Block	Condition × Block	Bonferroni pairwise comparisons between experimental conditions	B1	B2	B3
E1: Appeal	*F*(2,79)=19.19, *p*<.001, ηp^2^=.330	*F*(2,79)=0.41, *p*=.662, ηp^2^=.005	*F*(3.46, 136.83)=63.78, *p*<.001, ηp^2^=.620	High First vs Low First High First vs Random Low First vs Random	>.05 >.05 >.05	<.001 <.001 <.001	<.001 >.05 =.012
E2: Complexity	*F*(2,65)=1.49, *p*=.223, ηp^2^=.044	*F*(1.78,115.99)=0.95, *p*=.389, ηp^2^=.014	*F*(3.57, 115.99)=43.57, *p*<.001, ηp^2^=.573	High First vs Low First High First vs Random Low First vs Random	=.203 >.05 =.698	<.001 =.123 =.002	>.05 >.05 =.774
E3: Valence	*F*(2,77)=1.04, *p*=.359, ηp^2^=.026	*F*(2,154)=10.59, *p*<.001, ηp^2^=.121	*F*(4,154)=111.33, *p*<.001, ηp^2^=.743	High First vs Low First High First vs Random Low First vs Random	<.001 <.001 <.001	<.001 <.001 <.001	=.359 =.461 >.05
E4: Feelings	*F*(2,73)=1.69, *p*=.191, ηp^2^=.044	*F*(1.68,122.61)=2.69, *p*=.070, ηp^2^=.036	*F*(3.36, 122.61)=58.94, *p*<.001, ηp^2^=.617	High First vs Low First High First vs Random Low First vs Random	<.001 <.001 <.001	<.001 <.001 <.001	>.05 =.058 =.133
E5: Concreteness	*F*(2,71)=11.99, *p*<.001, ηp^2^=.253	*F*(1.34, 95.02)=.094, *p*=.831, ηp^2^=.001	*F*(1.34, 95.02)=80.48, *p*<.001, ηp^2^=.831	High First vs Low First High First vs Random Low First vs Random	<.001 =.713 <.001	<.001 <.001 <.001	=.001 =.389 =.045
E6: Semantic distance	*F*(2,64)=21.84, *p*<.001, ηp^2^=.406	*F*(1,49, 95.22)=13.33, *p*<.001, ηp^2^=.172	*F*(2.98,95.22)=215.60, *p*<.001, ηp^2^=.871	High First vs Low First High First vs Random Low First vs Random	<.001 <.001 <.001	<.001 <.001 <.001	<.001 >0.05 <.001

In all experiments, the Condition × Block interaction was highly significant and the effect sizes were large. Details for each experiment are reported below.

### Experiments 1 and 2: Visual icon characteristics

A similar pattern of findings was apparent for both visual appeal (E1) and complexity (E2), and so they are considered together here. In Block 1, participants in the High First condition were presented with only *highly appealing* or *highly complex* icons, while those in the Low First condition were shown only *very unappealing* or *very simple* icons, and those in the Random condition were shown a mixture of low-, mid-, and high-value icons. On the basis of the ratings from the pilot studies, one might therefore have expected high ratings in the High First condition, low ratings in the Low First condition, and mid-level ratings in the Random condition (see Fig. 2b). Bonferroni pairwise comparisons, however, showed that ratings did not differ between conditions in Block 1 (see Table 9, B1, for Experiments 1 and 2; cf. Fig. 2a with 3a and 3b, Block 1). This pattern of findings supports Hypothesis 1, that when the range of icons presented to participants is limited, they nevertheless provide ratings across the full range of the rating scale they are given, *biasing the ratings *obtained.

In Block 2 in the High First condition, participants viewed low-value icons, while those in the Low First condition viewed high-value icons. Bonferroni comparisons revealed that icons with lower values in Block 2 (in the High First condition) produced lower ratings in comparison to baseline, while those presented with higher-value ratings (in the Low First condition) produced much lower ratings (cf. Fig. 2a with 3a and 3b, Block 2; see also Table 9, B2). This pattern of findings supports Hypothesis 2, that changing the icon values presented to participants in the experimental conditions would change the rating context, providing a counter-nudge which produced a recalibration in participants’ ratings.

As expected, in Block 3, when the remaining 60 icons with mid-level values were presented in both High First and Low First experimental conditions, mean ratings returned to the mid-range and ratings became similar to those in the Random Baseline condition (see Table 9, B3).

### Experiments 3 and 4: Affective icon characteristics

For both valence (E3) and feelings (E4) ratings the patterns of findings in Block 1 approximates to that expected where ratings remained *unbiased* as a result of the range of stimuli presented (cf. Fig. 3c and d with Fig. 2b). In Block 1, participants shown high-value icons assigned high ratings (in the High First condition) while those shown low-value icons provided low ratings (in the Low First condition) in comparison with the Random Baseline (Table 9, B1). Nevertheless, as was the case for visual icon characteristics in Experiments 1 and 2, participants were very responsive to the change in icon sets as they moved from Block 1 to Block 2 (Table 9, B2). In the High First condition, ratings were much lower when low-value icons were presented in Block 2, and ratings were much higher when high-value icons were presented in Block 2. Participants in the experimental conditions provided ratings which did not differ from baseline in Block 3 (Table 9, B3).

## Experiments 5 and 6: Semantic icon characteristics

Findings were mixed for semantic icon characteristics. Ratings appeared to depend on the extent to which participants could rely on rating the visual appearance of items depicted in the icons as opposed to relying on accessing meaning.

### Experiment 5: Concreteness ratings

In the High First condition, participants appeared to rate concreteness on the basis of the items depicted in the icon and used the full range of ratings. As a result, no difference between the Random Baseline condition and High First condition emerged in Block 1 for concrete icons, but ratings adjusted accordingly when low-value and mid-value icons were shown in Blocks 2 and 3, respectively (cf. Fig. 3e with 3a and b where similar patterns are observed in the High First condition).

In contrast, when abstract icons were presented in Block 1 (i.e. low concreteness value icons), participants were more dependent on being able to access meaning from the icons (see Table 7 for examples of abstract icons), and as a result, ratings were lower in Block 1. In Block 2, ratings adjusted accordingly when concrete icons were presented (cf. Fig. 3e with 3c and d where similar patterns are observed in the Low First condition).

To sum up, the extent to which concreteness ratings are determined by the context provided by the range of icons in the set may depend on whether or not the icons are concrete, where participants may rely on the visual information in the icon, or abstract, where participants rely on the semantic concepts that are conveyed by the icon.

### Experiment 6: Semantic distance ratings

Semantic distance ratings were *unbiased* and did not change depending on the range of the stimuli shown. When high-value icons were presented, average ratings were high, when values were low, ratings were low, and the Random condition resulted in mid-value mean ratings. This reversed dramatically when low-value icons were presented in the High First condition and high-value icons were presented in the Low First condition in Block 2. Thus, ratings were not susceptible to nudge and bias as a result of limitations in the range of icon values presented (cf. Figs. 3f and 2b). The pattern of findings obtained in this experiment was also comparable to findings obtained in E3 and E4 (cf. Fig. 3f with 3c and 3d, see also Table 9, B1–B3).

## Discussion

Subjective ratings have played a critical role in the evaluation of stimulus characteristics of pictures and words, and the range of characteristics measured in this way has grown over time (e.g. Barry et al., 1997; Dan-Glauser & Scherer, 2011; Gilhooly & Logie, 1980; Ito et al., 1998; Paivio et al., 1968; Spreen & Schulz, 1966; Snodgrass & Vanderwart, 1980; Snodgrass & Yuditsky, 1996). This is also the case for icons (McDougall et al., 1999; Prada et al., 2015; Reppa et al., 2008; Rodrigues et al., 2018; Souza et al., 2020, 2021). The present series of experiments sought to examine potential bias in subjective ratings of the visual, affective, and semantic properties of icons. It was hypothesised that when the values of the to-be-rated icons was limited, this would change participants’ perceived choice architecture (Johnson et al., 2012; Thaler & Sunstein, 2008; Weinmann et al., 2016). The results suggested that bias occurred with some, but not all, ratings of icon characteristics. Bias occurred when ratings were based on *current visual experience* such as in Experiments 1 and 2, where participants rated the visual appeal or complexity of icons (see Fig. 3a and b). The similarity of the findings in E1 and E2 could not be attributed simply to an underlying correlation between visual icon characteristics, because appeal and complexity ratings were uncorrelated in the pilot studies (*r* = −.11, see Table 5). Participants’ ratings were much less amenable to bias when subjective judgements were based on *learned semantic associations*, i.e. semantic distance and the emotional attributions associated with icons in Experiments 3 and 4 (see Fig. 3c and d).

It is worth considering the nature of the judgements being made in these experiments to understand why learned associations may be an important determiner of ratings and serves to protect again nudge and bias. Participants rating icon valence in E3, for example, were asked to make judgements about the positivity or negativity of the icons. For this task, the meaning associated with the icons was critical. Consider rating valence of icons such as ‘dental examination’ and ‘tsunami’ versus ‘arriving flights’ and ‘thumbs up’. Similar kinds of judgements were required when rating ‘feelings’ in E4. The extent to which icons might be regarded as depressing or cheery are likely to be made on the basis of existing associations (e.g. when rating the ‘virus’/’low battery’ versus ‘package’/’celebration’ icons shown in Table 7). Ratings of semantic distance, the goodness of fit between what is shown in the icon visually and its intended meaning, also relied on associations between what was depicted in the icon and its given meaning learned over time: consider ‘baggage reclaim’/’campfire’ versus rating ‘line vessel’ or ‘pixel averaging’ (see Table 7).

A mixed picture emerged for concreteness ratings. Concrete icons are expected to contain known visual entities, i.e. real things, people, or materials, while abstract icons do not, instead consisting of shapes and lines (see rating instructions for concreteness in Table 8). Judgements of concreteness commonly require judgements of ease of visual object recognition, while judgements about abstract, non-pictorial icons require more evaluation of the possible associations (or lack of them) with known meaning. Consider rating the concrete icons such as zoo and cycling route shown in Table 7. Visual recognition is sufficient to render the item ‘concrete’ as opposed to abstract icons such as newsfeed and half-width widening (the two low-concreteness, or abstract, items in Table 7), where objects, if any, are difficult to identify and a search for possible meaning already associated with the icon is needed. Taken together across all six experiments, the results from Block 1 suggest that judgements of visual and semantic object attributes are sensitive to contextual nudge, which biases ratings. Interestingly, affective ratings (valence and feelings) and semantic distance ratings show little evidence of being susceptible to the contextual range in icon values.

The pattern of results in Blocks 2 and 3 reveals the dynamic nature of participants’ icon perceptions and how quickly and effectively they adapted and recalibrated their ratings when a fuller range of icon values became apparent, i.e. they were susceptible to counter-nudge. In Block 2, participants were either shown low-value icons having seen high-value icons previously (the High First condition) or shown high-value icons having seen low-value icons previously (the Low First condition). In both conditions, the pattern of ratings changed almost immediately: in the High First condition, ratings became much lower in Block 2, and in the Low First condition, ratings became much higher. The source of bias—the assumption that participants were seeing the full range of icon values in Block 1—was lost, and participants’ ratings closely followed the experimental manipulations (high ratings for high-value icons and low for low-value icons). This occurred despite the fact that participants were never made aware of any change in the nature of the icons being presented. Participants’ sensitivity to change in the nature of the icon set was confirmed in Block 3, where those in the High First and Low First conditions were shown mid-level icons, and the mean ratings of participants in both conditions returned to those mid-levels.

In the choice architecture literature, a sometimes implicit assumption is made that nudge operates by altering an individual’s stimulus environment in such a way as to trigger automatic cognitive processes which then change behaviour. The evidence from our study suggests that nudges which catalyse bias, or change, in participants’ ratings may take different forms. Two nudge processes appeared to be operating in the current study. One nudge operated when the range of stimuli presented in Block 1 was limited, and this created bias as a result of *participants’ expectation* that a full range of icon values was being presented, resulting in the full range of ratings being employed despite the constrained nature of the stimuli being presented, and so ratings bias occurred. However, this nudge was only effective when participants’ ratings judgements relied on current visual experience rather than learned semantic associations. A second nudge operated when participants moved from Block 1 through to Blocks 2 and 3 (see Fig. 3). Participants rapidly recalibrated their ratings as the range of stimuli changed from one block to another. The changes in the ‘stimulus environment’ created *cognitive contrasts* which automatically and unconsciously changed participants’ behaviour. Considerable care is needed in creating appropriate stimulus environments for subjective rating of icon stimuli.

Given these findings, should we, as Forsythe et al. (2017) suggested, replace subjective ratings with metrics which may be more predictable and reliable than individuals’ perceptions? Our evidence suggests that this depends on which icon characteristics are important for the research being carried out and/or the practical application to which icons are being put. Mandera, and Keuleers, and Brysbaert, M. (2015) suggest that creating new metrics to replace subjective ratings may not always be straightforward. They carried out one of the few systematic studies examining possible prediction of word/lemma ratings from existing corpora using several automatic, or metrics-based, techniques. The concreteness, age of acquisition, arousal, dominance, and valence of the stimuli were predicted with varying success: for arousal, dominance, and valence, the correlations with the original ratings were relatively poor. Mandera et al. concluded that even if the correlation between the original and the extrapolated ratings created using the new automated metrics was high, there was always a part of the variance that remained unexplained, and that, depending on the methods used, such methods may introduce a range of other artefacts to the extrapolated rating data.

Forsythe et al.’s arguments should not be considered out of hand, however, when one considers the small number of icon ratings corpora currently available for use by researchers or designers. Until relatively recently, creating new icon sets was not only time-consuming but often depended on technical experts who may not necessarily have design expertise. Design has been improved by the availability of online resources such as the International Organization for Standardization ISO 7000 database for graphical symbols (n.d), The Noun Project (n.d.), a large online resource of icons and pictures, and also Google’s Material Design Iconography section (Google Design Guidelines, n.d.). Further work is needed, similar to that undertaken by Mandera et al., to examine how ratings might be created using automated metrics or, alternatively, crowdsourcing could be used to obtain online participant ratings of large corpora and, importantly, employ more diverse participant groups across cultures, countries, social backgrounds, ages, and genders. Where metrics are not available, or are likely to be a poor substitute for ratings, we need to understand how, and under what conditions, subjective ratings may be obtained and utilised reliably without bias.

There are important practical implications of these findings, particularly for icon evaluation and especially if icons are likely to be used in time- and safety-critical applications. Icon sets for evaluation need to vary sufficiently on key characteristics and need to be sufficiently large, containing sufficient ‘distractors’ as well as the ‘target’ items being considered for use. Even with these better design resources and further ratings corpora, the icons chosen for evaluation are often those regarded as the best fit for subsequent use. Ironically, if the icons being rated are well designed, this might have the effect of *under*-valuing their potential usefulness when the critical icon characteristics are visually based. In the High First conditions in Experiments 1 and 2 where high-value novel icons were presented, nudge and bias produced a lower mean rating (of approximately 4 as opposed to 6). Just as importantly, if icons are poorly designed, the presentation of a limited range of icons with low values for rating may *over*-value their potential. In the Low First conditions in Experiments 1 and 2, low-value novel icons were presented, creating bias in ratings and producing higher mean ratings than might otherwise have been expected (ratings of approximately 4 as opposed to 2). This could lead to hazards where icons are used in contexts which are time- or safety-critical, when icon search times may be affected by both visual complexity and appeal (e.g. icons used in heads-up cockpit displays or chemical/nuclear plant monitoring; McDougall et al., 2000, 2006; Reppa & McDougall, 2015).

The practical implications of the current study may be less apparent for psycholinguistic stimuli. As already noted, researchers using psycholinguistic stimuli are aware of the need to employ large representative sets when carrying out norming studies. However, potential bias may result once stimuli have been selected for experimentation and constrained by the need for experimental control. As Clark (1973) pointed out, such selection may result in a sample of materials which are not representative of the original, larger, set. While Clark and others have provided statistical solutions to this lack of representativeness, such solutions do not deal with potentially different patterns of responding by participants when materials are constrained. Our study does not provide evidence with regard to this issue, but it does suggest that it may be worth examining in the future, particularly where visual judgements of stimuli are in focus.

Finally, a degree of caution is required when considering these findings, not least because this is the first time, to our knowledge, that the dynamic nature of ratings bias for icons has been demonstrated. Replication is needed, particularly for icon concreteness. The experimental design necessarily precluded consideration of more than one icon characteristic, but we know from previous research that icon characteristics together have a synergistic effect on performance (Forsythe et al., 2008, 2017; Reppa et al., 2021; Reppa & McDougall, 2015), and icon characteristics vary in importance as icons become visually familiar and icon–function relationships are learned (Forsythe et al., 2008; McDougall & Isherwood, 2009; McDougall et al., 2000). Other important stimulus characteristics such as colour and trust also need to be considered in future research, since very little systematic research has examined these important icon characteristics (but see Horton, 1994; Liu et al., 2021; Richardson, Revell, Kim, et al., 2021). Similarly, there is a growing need for better understanding of emojis, which are increasingly used in online media including quantification of their particular characteristics (see Daniel & Camp, 2020; Fischer & Herbert, 2021; Rodrigues et al., 2018). Despite these caveats, the potential bias resulting from the rating context in subjective ratings of icons is clear and should be noted by icon designers and researchers alike, particularly where time-critical or safety-critical icons are being evaluated.
